# The Association between Intracranial Calcifications and Symptoms in Patients with Primary Familial Brain Calcification

**DOI:** 10.3390/jcm13030828

**Published:** 2024-01-31

**Authors:** Gini Mathijssen, Evelien van Valen, Pim A. de Jong, Nienke M. S. Golüke, Emiel A. van Maren, Birgitta M. G. Snijders, Eva H. Brilstra, Ynte M. Ruigrok, Susan Bakker, Renzo W. Goto, Marielle H. Emmelot-Vonk, Huiberdina L. Koek

**Affiliations:** 1 Department of Geriatrics, University Medical Center Utrecht, Utrecht University, Heidelberglaan 100, 3584 CX Utrecht, The Netherlands; g.mathijssen-3@umcutrecht.nl (G.M.); n.m.s.goluke@umcutrecht.nl (N.M.S.G.); r.w.goto@umcutrecht.nl (R.W.G.); m.h.emmelotvonk@umcutrecht.nl (M.H.E.-V.); 2 Department of Radiology, University Medical Center Utrecht, Utrecht University, Heidelberglaan 100, 3584 CX Utrecht, The Netherlands; p.dejong-8@umcutrecht.nl (P.A.d.J.); e.a.vanmaren@umcutrecht.nl (E.A.v.M.); 3 Department of Geriatrics, Hospital Gelderse Vallei, Willy Brandtlaan 10, 6716 RP Ede, The Netherlands; 4 Department of Genetics, University Medical Center Utrecht, Utrecht University, Heidelberglaan 100, 3584 CX Utrecht, The Netherlands; e.h.brilstra@umcutrecht.nl; 5 Department of Neurology and Neurosurgery, University Medical Center Utrecht, Heidelberglaan 100, Utrecht University, 3584 CX Utrecht, The Netherlands; ij.m.ruigrok@umcutrecht.nl; 6 Department of Rehabilitation, Physical Therapy Science & Sports, University Medical Center Utrecht, Utrecht University, Heidelberglaan 100, 3584 CX Utrecht, The Netherlands; s.bakker-8@umcutrecht.nl

**Keywords:** primary familial brain calcification, PFBC, Fahr’s disease, total calcification score, cognitive disorders, motor dysfunction

## Abstract

(1) Background: Primary Familial Brain Calcification (PFBC) is a neurodegenerative disease characterized by bilateral calcifications of the basal ganglia and other intracranial areas. Many patients experience symptoms of motor dysfunction and cognitive disorders. The aim of this study was to investigate the association between the amount and location of intracranial calcifications with these symptoms. (2) Methods: Patients with suspected PFBC referred to our outpatient clinic underwent a clinical work-up. Intracranial calcifications were visualized on Computed Tomography (CT), and a Total Calcification Score (TCS) was constructed. Logistic and linear regression models were performed. (3) Results: Fifty patients with PFBC were included in this study (median age 64.0 years, 50% women). Of the forty-one symptomatic patients (82.0%), 78.8% showed motor dysfunction, and 70.7% showed cognitive disorders. In multivariate analysis, the TCS was associated with bradykinesia/hypokinesia (OR 1.07, 95%-CI 1.02–1.12, *p* < 0.01), gait ataxia (OR 1.06, 95%-CI 1.00–1.12, *p* = 0.04), increased fall risk (OR 1.04, 95%-CI 1.00–1.08, *p* = 0.03), and attention/processing speed disorders (OR 1.06, 95%-CI 1.01–1.12, *p* = 0.02). Calcifications of the lentiform nucleus and subcortical white matter were associated with motor and cognitive disorders. (4) Conclusions: cognitive and motor symptoms are common among patients with PFBC, and there is an association between intracranial calcifications and these symptoms.

## 1. Introduction

Primary Familial Brain Calcification (PFBC), also known as Fahr’s disease or idiopathic basal ganglia calcification, is a neurodegenerative disease characterized by bilateral brain calcifications, predominantly located in the basal ganglia. Other areas of the brain can also be affected, such as the thalami, subcortical white matter, cerebral cortex, and cerebellar hemispheres [1,2]. The estimated prevalence of PFBC varies between 0.45 and 2.1 per 1000 [3], but it could be as high as 6.6 per 1000 [4]. PFBC often has an autosomal dominant pattern of inheritance, with gene mutations in *SLC20A2*, *PDGFB*, *PDGFRB,* and *XPR1* [5,6,7,8]. Furthermore, mutations in *MYORG* and *JAM2* have been reported to cause PFBC with an autosomal recessive pattern of inheritance [9,10]. In almost 50% of the affected patients, the gene mutation remains unknown [11,12].

PFBC is clinically heterogeneous, and patients may present with a wide variety of symptoms, including movement dysfunction (mostly akinetic–hypertonic syndrome), cognitive disorders, and neuropsychiatric disorders [1,2,13,14]. When present, clinical symptoms of PFBC range from mild to severely disabling [15]. In addition, persons with proven calcium deposits and genetically confirmed PFBC can be asymptomatic [1,14]. For this reason, PFBC is mostly diagnosed based on computed tomography (CT) in the absence of other conditions that cause intracranial calcifications. Matching clinical symptoms, a positive family history of PFBC, and a known genetic mutation further support or confirm the diagnosis [4,11,14]. Other conditions that may cause intracranial calcifications include endocrine disorders like hypoparathyroidism and pseudohypoparathyroidism [16,17], neurotoxins (e.g., lead poisoning), and infectious diseases [18]. If the basal ganglia calcifications are secondary to another cause, the term Fahr’s syndrome is often used. Basal ganglia calcifications can occur as a process of aging, with a prevalence of up to 30% in older adults [19,20]. However, these calcifications are often less severe and less diffusely distributed throughout the brain compared to patients with PFBC [14,21].

The association between the amount and location of intracranial calcifications and clinical symptoms remains largely unknown. A recent study in a more general population found no association between basal ganglia calcifications and cognitive function in patients referred to a memory clinic [22]. In addition, a systematic review concluded that no obvious association was found between the location of calcification and the type of clinical signs observed in patients with PFBC [15]. In contrast, several studies with PFBC have shown that symptomatic patients have significantly more brain calcifications than asymptomatic patients [2,13,14,21,23]. Therefore, the aim of this study was to further explore the association of the amount and location of intracranial calcifications with motor dysfunction and cognitive disorders in a consecutive sample of patients with PFBC.

## 2. Materials and Methods

### 2.1. Study Population

The study population consisted of all patients aged 18 years and above with suspected PFBC who were recruited between September 2019 and June 2023 from the geriatric outpatient clinic of the University Medical Centre Utrecht, the Netherlands. For the present study, the following criteria were used to diagnose PFBC: (a) Bilateral calcifications of the basal ganglia visualized on a CT scan that could not be explained by normal aging. Other brain regions could also be affected; and (b) no secondary cause was retrieved after extensive etiological assessment, including metabolic, infectious, toxic, or traumatic causes of calcifications. Supportive criteria were (c) clinical symptoms consistent with PFBC and (d) a family history consistent with genetic inheritance [1,17,23]. A known genetic mutation (*PDGFB*, *PDGFRB*, *SLC20A2*, *XPR1*, *MYORG,* or *JAM2*) confirmed the diagnosis [10,11,12]. Patients who did not meet the aforementioned criteria or whose calcifications were caused by a secondary cause were excluded from analyses. This study was part of an ongoing prospective cohort study investigating PFBC. The data presented here were obtained at the baseline clinical assessment. Ethical approval was waived by the Dutch local Medical Research Ethics Committee Utrecht (registration number 21-170).

### 2.2. Diagnostic Procedures

All patients underwent an extensive clinical work-up during their first visit to the geriatric outpatient clinic. A multidisciplinary team conducted assessments for all patients. A geriatrician, radiologist, nurse, geneticist, rehabilitation physician, neuropsychologist, and physiotherapist were part of the multidisciplinary team. A neurologist was also part of the research group but not routinely involved in evaluating the patients. However, the neurologist was available for consultation if necessary. The assessment consisted of a medical history, medication review, (hetero)anamnesis, vital signs, general physical, neurological and psychiatric examination, and neuropsychological assessment. For all patients, the age at baseline visit and level of education were noted. The level of education was classified according to the Verhage education scale [24]. Secondary causes of brain calcifications were excluded by extensive laboratory and serological testing (see Appendix A, Table A1 for an overview). A Computed Tomography (CT) scan of the brain was the standard imaging performed, as (smaller) calcifications are more readily detected on CT compared to other imaging modalities like magnetic imaging (MR). Genetic analysis was performed on all patients who gave informed consent. Whole exome sequencing was performed, followed by variant analysis of 6 genes that have been associated with PFBC (*PDGFB, PDGFRB, SCL20A2, XPR1, MYORG,* and *JAM2*).

The clinical diagnosis regarding cognition (i.e., subjective cognitive decline, MCI, or dementia) was based on the Dutch national multidisciplinary guideline on diagnostics and treatment of dementia [25,26]. MCI and dementia correspond with the DSM-5 minor neurocognitive disorder and major neurocognitive disorder, respectively [27]. A patient was classified as having subjective cognitive decline when a patient reported cognitive complaints, but no cognitive disorders were objectified during neuropsychological assessment. MCI was defined as (I) cognitive complaints as reported by the patient or informant; (II) evidence from the neuropsychological assessments indicating a disorder in one or more cognitive domains with a decline compared to the individual’s previous level of functioning; and (III) no significant interference with complex daily life activities. A patient was defined as having dementia in the presence of significant interference with complex daily life activities [25].

#### 2.2.1. Motor Dysfunction

Motor dysfunction was evaluated by both the geriatrician and the physiotherapist as part of a comprehensive physical examination. The geriatrician and physiotherapist had extensive expertise in diagnosing and treating patients with parkinsonism, including patients with PFBC. In this study, primary emphasis was placed on the analysis of Parkinsonian symptoms, cerebellar dysfunction, and increased fall risk. Following the MDS Clinical Diagnostic Criteria for Parkinson’s Disease, a combination of bradykinesia/hypokinesia plus rest tremor or rigidity or both was defined as Parkinsonism [28]. Bradykinesia/hypokinesia, rigidity, and type of tremor (rest, postural, or intention tremor) were individually recorded as being either “absent” or “present”. Cerebellar dysfunction was defined as the presence of either limb ataxia, gait ataxia, or intention tremor, or a combination of these symptoms. Additionally, increased fall risk was assessed using the 28-point version of the Performance-Oriented Mobility Assessment [29], the Dynamic Gait Index [30], and the Mini Balance Evaluation Systems Test (Mini-BESTest) [31]. A Performance-Oriented Mobility Assessment total score below 19 [32], a Mini-BESTest score below 19 [33], or a Dynamic Gait Index score of 19 or below were defined as having an increased fall risk [34]. If a patient was recorded as having Parkinsonism, cerebellar dysfunction, and/or if a patient was defined as having an increased fall risk, the patient was classified as symptomatic. To further explore Parkinsonian features, the motor subscale (part III) of the Unified Parkinson’s Disease Rating Scale (UPDRS) was employed [35]. The UPDRS is a clinical rating scale with no given clinical cut-off score for defining motor symptoms. Therefore, the UPDRS score was treated as a continuous variable in the analysis.

#### 2.2.2. Cognitive Disorders

Cognitive disorders were objectified during a neuropsychological evaluation performed by a trained neuropsychologist. Global cognitive functioning was evaluated using the Montreal Cognitive Assessment (MoCA) [36,37]. Global cognitive functioning was considered impaired when the MoCA score was below 26 [37]. In addition, a comprehensive neuropsychological assessment was administered, representing three cognitive domains often affected in PFBC, including attention/processing speed, executive function, and memory [14,38] (see Table 1 for test details). For differential diagnostic purposes, additional neuropsychological tests were administered when necessary. Tests that were completed by at least 80% of all patients were included in the analysis. Raw test scores were corrected for age and level of education and transformed to T-scores upon normative data available via the Advanced Neuropsychological Diagnostics Infrastructure (ANDI Norms 2.0.6, update 13-07-2021) [39]. As per our standard clinical practice, a cognitive disorder can be confirmed when a patient demonstrates impaired performance on a minimum of two tests within a specific cognitive domain [40]. In the context of this study, composite scores were computed of performance in each domain for each patient, by averaging the standardized individual test scores within a domain for each patient. Patients who completed less than two tests within a cognitive domain were excluded from analysis on that domain. A composite score below T33 was considered abnormal (percentile 5) and defined as a cognitive disorder. If the patient had at least one cognitive domain disorder or if global cognitive functioning was impaired, the patient was classified as being symptomatic.

### 2.3. Calcification Measurements

To quantify the amount of calcium deposits on CT, we used the total calcification score (TCS) as described by Nicolas et al. [14]. The severity of calcification was graded from 0 (no calcification) to 5 (severe and confluent calcification) in the following locations: left and right lentiform nucleus, left and right caudate nucleus, left and right thalamus, left and right cerebral subcortical white matter, cerebral cortex, left and right cerebellar hemisphere, vermis, left and right midbrain, pons, and medulla. The left and right internal capsule was only scored if independent of other calcifications. Adding up the individual scores would lead to a TCS between 0 and 80. All CT scans were scored according to this method by a board-certified radiologist with a special interest and expertise in PFBC, blinded for clinical variables.

### 2.4. Statistical Analysis

The baseline characteristics were analyzed using descriptive statistics. Continuous variables were reported as the median and range. Categorical variables were reported as a number with the corresponding percentage of the total group size unless otherwise stated. Outcomes were dichotomized into the presence of motor dysfunction and the presence of cognitive disorders according to the aforementioned definitions.

Logistic regression models were used to investigate the association between the total amount of calcifications and the amount of calcifications in the earlier described brain areas and the presence of motor dysfunction and cognitive disorders. Univariate and age and sex-adjusted odds ratios (OR) with a 95% confidence interval (CI) were calculated. A multivariate linear regression model was performed to investigate the influence of the total amount of calcifications and age on the UPDRS score, with the total amount of calcifications and age as independent variables.

The IBM SPSS Statistics version 28.0.1.0 (SPSS Inc., Chicago, IL, USA) was used for the data analyses. A *p*-value < 0.05 was considered statistically significant.

## 3. Results

### 3.1. Demographic and Clinical Characteristics

**Figure A1:**
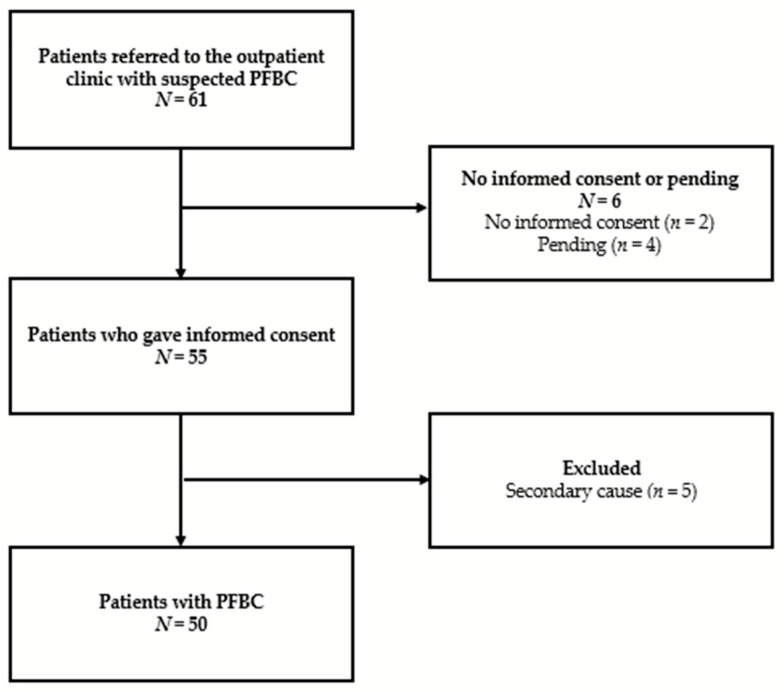
Flowchart of the study population. PFBC = Primary Familial Brain Calcification.

Of the 61 eligible persons referred to our outpatient clinic with suspected PFBC, 6 patients did not give informed consent to participate in the study. Of the 55 patients who gave informed consent, 5 patients did not meet the aforementioned criteria for PFBC and were therefore excluded from the analysis. The reason for exclusion was a diagnosis of hypoparathyroidism in four patients and pseudohypoparathyroidism in one patient (see Appendix B, Figure A1, for a flowchart of the study population). The 50 patients that were included in the study population had a median age of 64.0 years (age range from 18 to 88 years). An equal number of men and women were included (25 women, 50%). Two subjects were from the same family. Genetic analysis was made available to all patients, and 41 patients underwent the analysis upon their request. Of the 35 patients for whom genetic analysis was completed, PFBC-associated gene mutations were found in almost half of the patients (48.6%), with mutations within *SLC20A2* as the most common outcome (Table 2). Upon the first visit to our clinic, most patients were independent in activities of daily living (94.0%). Of the 50 patients, 82.0% were symptomatic according to the aforementioned definitions. Descriptive data are shown in Table 2.

#### 3.1.1. Motor Dysfunction

As shown in Table 2, the majority of the symptomatic patients were classified as having motor dysfunction (78.0%). Of the patients with motor dysfunction, cerebellar dysfunction was observed in 25 patients (78.1%), an increased fall risk was observed in 16 patients (50.0%), and 11 patients met the criteria for Parkinsonism (34.4%).

Bradykinesia/hypokinesia was observed in 15 patients in total (46.9% of the patients with motor dysfunction). Four patients showed bradykinesia/hypokinesia without rest tremor or rigidity (12.5%). Tremor was observed in 14 patients (43.8%), with a rest tremor in 7 patients, intention tremor in 4 patients, postural tremor in 2 patients, and for 1 patient, the tremor type was unspecified. Rest tremor without bradykinesia/hypokinesia and rigidity was observed in three patients (9.4%). Rigidity without bradykinesia/hypokinesia and rest tremor was observed in three patients (9.4%).

Of the cerebellar signs, limb ataxia was found in 21 patients (65.6%), of whom 14 patients were without gait ataxia or intention tremor (43.8%). Gait ataxia and intention tremor were observed in eight patients (25.0%) and four patients (12.5%), respectively. Two patients (6.3%) had gait ataxia, and similarly, two patients had intention tremor (6.3%) in the absence of other cerebellar signs. Nine patients showed more than one cerebellar symptom (28.1%).

#### 3.1.2. Cognitive Disorders

Two patients were excluded from cognitive analysis due to performance below threshold on performance validity testing during the neuropsychological assessment. The immediate recall of the Rey–Osterrieth Complex Figure Test, the Digit Symbol Substitution Test of the Wechsler Adult Intelligence Scale IV (WAIS-IV-NL), and the Letter Fluency Test were completed by less than 80% of patients and were therefore excluded from the composite score. Reasons for not administering the complete test battery were mostly due to practical considerations (mostly time restraints) or patient characteristics (mostly fatigue). One patient completed less than two tests within the memory domain and was therefore excluded from analysis on that domain. Similarly, one patient completed less than two tests within the executive function domain.

Of the 48 patients, half had an impairment in global cognitive functioning (median score 25; range 12–29). Table 2 shows that of the patients with cognitive disorders, 12 patients had disorders in the domain of attention/processing speed (42.9%). Less often affected were memory (in six patients, 21.4%) and executive function (in four patients, 13.8%).

### 3.2. Brain Calcifications

The mean TCS was 29.0 (95%-CI 26.4–31.6, range 4–66). Calcifications of varying severity were widespread among patients, and the most prevalently involved site was the lentiform nucleus (96% of all patients), as shown in Table 3. In terms of severity per brain area, the lentiform nucleus calcium deposits were not only the most commonly found but also the most severe (mean 7.8, SD 2.5). Other areas frequently affected and with notable calcifications were the cerebellar hemispheres (dentate nucleus) in 78% of patients (mean 4.8, SD 3.6), the caudate nucleus in 70% (mean 4.8, SD 4.3), and thalamus in 68% (mean 4.8, SD 3.8). The intracranial areas that were the least common for brain calcifications were the midbrain, pons, and medulla. These areas also showed the least severe calcifications.

### 3.3. Association between the Total Amount of Calcifications and Motor and Cognitive Disorders

Results of the uni- and multivariate logistic regression analysis are presented in Table 4. Multivariate analysis showed a significant association between the TCS and increased fall risk (OR: 1.04, 95%-CI 1.00–1.08, *p* = 0.03) and between the TCS and bradykinesia/hypokinesia (OR 1.07, 95%-CI 1.02–1.12, *p* < 0.01). No significant association was found between the TCS and the presence of rest tremor or rigidity. Among the symptoms associated with cerebellar dysfunction, the multivariate analysis showed a significant association between the TCS and gait ataxia (OR: 1.06, 95%-CI 1.00–1.12, *p* = 0.04). In the multivariate analysis, no significant association was found between the TCS and limb ataxia or intention tremor.

In addition, the multivariate analysis showed a significant association between the TCS and attention/processing speed (OR: 1.06, 95%-CI 1.01–1.12, *p* = 0.02). No significant association was found for the other cognitive domains.

The multivariate linear regression analysis showed a significant association between the UPDRS and the TCS (R^2^ = 0.33, F(2, 43) = 12.22, *p* < 0.01).

### 3.4. Association between Location of Calcifications and Motor Dysfunction and Cognitive Disorders

Multivariate analysis showed that the presence of motor dysfunction was associated with calcifications of the lentiform nucleus (OR: 1.50, 95%-CI 1.07–2.09, *p* = 0.02). Calcification of the subcortical white matter in the multivariate model was associated with the presence of cognitive disorders (OR: 1.25, 95%-CI 1.01–1.54, *p* = 0.04). No significant associations were found for the other locations; see Table 5.

## 4. Discussion

The aim of this study was to explore the association of the amount and the location of intracranial calcifications with motor dysfunction and cognitive disorders in patients diagnosed with PFBC. This study found that both motor dysfunction and cognitive disorders are common among patients with PFBC. The total amount of calcifications was significantly associated with bradykinesia/hypokinesia, gait ataxia, and increased fall risk. Additionally, the total amount of calcifications was significantly associated with disorders in attention/processing speed. Calcifications in the lentiform nucleus were associated with motor dysfunction. Subcortical white matter calcifications were associated with the presence of cognitive disorders.

The distribution and severity of intracranial calcifications observed in this study align with previous research employing the same visual rating scale [2,14]. The frequency at which the various motor disorders and cognitive disorders occurred within this study population is predominantly consistent with findings from several other studies. To illustrate, Manyam et al. described the presence of movement disorders in 55% of patients [13], compared to 64% in our study cohort (78% of our symptomatic population). Similarly, Nicolas et al. found movement disorders in 54.9% of all patients and cognitive disorders in 58.8% of all patients [14], compared to the prevalence of 58% of cognitive disorders in our study population (71% of our symptomatic population). In a later study by Nicolas et al., cognitive disorders were found in up to 64.7% of all patients, and movement disorders in up to 76.5% [2]. The observed differences between studies can be explained by a couple of factors. First, it is imperative to emphasize that the current study focuses on a subset of frequently observed motor symptoms, thus omitting others. Also, the diverse definitions of motor and cognitive symptoms or disorders across studies, as well as the variations in neurological, physiological, and neuropsychological examinations, make detailed comparisons difficult. To illustrate, most assumptions about cognition in patients with PFBC are based on symptom reports rather than based on objective findings from neuropsychological evaluations [1,2,4,13,23,53,54]. In addition, what is considered a cognitive symptom is often not well defined [11,18]. This highlights the need for more standardized methods to assess these symptoms and disorders in both research and clinical practice. Given the significant correlation between the total amount of calcifications and the UPDRS score, it is plausible to consider this assessment as a viable option for future research on motor symptoms in PFBC.

Notable differences exist in the previously reported cognitive profile of patients with PFBC compared to our findings. In most studies, the cognitive profile predominantly comprised disorders in memory and executive function [2,14,38,55]. The comparatively less prominent occurrence of disorders in executive function and memory in our study could potentially be attributed to a couple of factors. First, the results from the executive function tests included in this study were all adjusted for processing speed, preventing scores from being classified as impaired solely due to the often slow processing speed observed in this patient population. Secondly, only scores that were in the lowest 5% range compared to the normative data were considered impaired. Lastly, in our study, cognitive disorders were based on impaired scores on at least two neuropsychological tests within one domain, as preferred in standard clinical practice [40]. These factors could have led to a more robust estimation of the cognitive disorders compared to the previous studies.

It is worth noting that in our study, the criteria for cognitive disorders were stringent to ensure high specificity. Hence, more subtle cognitive changes (e.g., standardized individual test scores below average) that might be clinically relevant are not considered in this study. For the assessment of motor dysfunction such as increased fall risk, measurements and cut-off scores were employed that are routinely used in geriatric clinical practice. However, balance scores decline with age, and the presently employed cut-off scores may be overly stringent for a younger patient population, such as those with PFBC [56]. In routine patient care, one might opt for less stringent criteria to prevent overlooking motor and cognitive decline that could significantly impact independence in the activities of daily living and quality of life.

A strength of this study is that we extensively assessed a relatively large cohort of patients with PFBC using standardized and validated measures. Furthermore, cognitive disorders were well defined and assessed in compliance with clinical practice using a wide variety of tests for global cognitive functioning and different cognitive domains. The results of this study therefore have high internal and external validity and provide clear-cut definitions of motor and cognitive disorders that can be used for future research on PFBC.

This study has certain limitations. While the cohort size was relatively large, considering that PFBC is a rare disease, it remained constrained. Consequently, not all cognitive and motor symptoms (e.g., visuo-constructive disorders and other hyperkinetic movement disorders) could be evaluated, and a limited number of confounders could be included in the analysis. Additionally, some data was missing (e.g., for the UPDRS and cognitive assessment), attributed to the observational design of this study. Furthermore, videotaping of the movement disorders and subsequent analysis of the recordings by a movement disorders specialist was not performed. Therefore, it is possible that symptoms of movement disorders were missed or misclassified, particularly if they were subtle. Lastly, a genetic mutation was not found in about half of the patients who underwent genetic testing. This is consistent with other studies [2], and it is conceivable that pathogenic changes in several additional genes will be identified in the future [12]. The calcifications of the patients with no mutation found or with no genetic testing could still have been caused by a secondary cause, even though extensive laboratory testing did not show abnormalities in these patients.

Future longitudinal research is needed to further confirm the association between the amount of calcifications and the expression of symptoms and progression over time in patients with PFBC. In addition, more in-depth research on the cognitive profile of patients is necessary to accurately identify more subtle, early cognitive changes that are already frequently reported by patients. Finally, there is a need for future longitudinal research on psychiatric symptoms. These nonmotor symptoms frequently manifest in patients with PFBC [14,23,38,57,58], and the available research on this topic is scarce.

Ultimately, more knowledge on the progression over time of the calcifications and symptoms will open up the possibility of giving patients a more accurate prognosis than is currently possible. Also, more knowledge is needed about the best-matched psychological and physiotherapeutic interventions during the disease course, as there is no cure for the disease yet.

## 5. Conclusions

In summary, this study showed that both motor dysfunction and cognitive disorders are common among patients with PFBC. The total amount of calcifications was associated with bradykinesia/hypokinesia, gait ataxia, increased fall risk, and with cognitive disorders in attention/processing speed. Calcifications of the lentiform nucleus and subcortical white matter were associated with motor dysfunction and cognitive disorders, respectively. Large cohort studies are needed to better understand this clinical and genetic heterogeneous disease.

## Figures and Tables

**Table 1:** Neuropsychological tests.

Global Cognitive Functioning
Montreal Cognitive Assessment [36,37]
Attention/Processing speed
Trail Making Test part A [41,42]
Stroop Test reading and color naming [43]
WAIS-IV-NL Digital Symbol Substitution Test [44]
WAIS-III-NL Forward Digit Span [45]
Executive function
Trail Making Test part B (adjusted for part A) [41,42]
Stroop Test color–word interference [43]
WAIS-III-NL Backward Digit span [45]
Letter fluency test [46]
GIT-2 semantic fluency test [47]
Memory ^1^
MoCA Memory Index Score [36]
Rivermead Behavioral Memory Test immediate and delayed recall [48,49]
15-word Auditory Verbal Learning Test immediate and delayed recall [50]
Rey-Osterrieth Complex Figure Test immediate and delayed recall [51]
Visual Association Test [52]

Note. WAIS = Wechsler Adult Intelligence Scale; GIT-2 = Groninger Intelligentietest 2; ^1^ Verbal memory was assessed either by the Rivermead Behavioral Memory Test or the Auditory Verbal Learning Test.

**Table 2:** Demographic and clinical characteristics (*N* = 50).

Variable	*N* (%)
Female	25 (50.0)
Educational level ^1^	
Low (Verhage 1–4)	14 (28.0)
Average (Verhage 5)	15 (30.0)
High (Verhage 6–7)	21 (42.0)
Family history ^1^	
PFBC	11 (22.0)
Parkinson/Parkinsonism (*n* = 49)	3 (6.0)
Dementia (*n* = 49)	18 (36.0)
Psychiatric disorders (*n* = 44)	12 (24.0)
Activities of daily living ^1^	
ADL independent	47 (94.0)
iADL independent	37 (74.0)
Genetic testing ^1^	41 (82.0)
No genetic mutation	18 (43.9)
Results not known yet ^2^	6 (14.6)
Genetic mutation ^2^	17 (41.5)
*SLC20A2* ^3^	10 (58.8)
*XPR1* ^3^	3 (17.6)
*PDGFB* ^3^	2 (11.8)
*MYORG* ^3^	2 (11.8)
*PDGFRB* ^3^	0 (0.0)
*JAM2* ^3^	0 (0.0)
Symptomatic ^1^	41 (82.0)
Motor dysfunction ^4^	32 (78.0)
Parkinsonism ^5 6^	11 (34.4)
Bradykinesia/hypokinesia ^6^	15 (46.9)
Rest tremor ^6^	7 (21.9)
Rigidity ^6^	14 (43.8)
Cerebellar dysfunction ^6 7^	25 (78.1)
Limb ataxia ^6^	21 (65.6)
Gait ataxia ^6^	8 (25.0)
Intention tremor ^6^	4 (12.5)
Increased fall risk ^6^	16 (50.0)
Cognitive disorders ^4 8^	29 (70.7)
Global cognitive functioning impaired ^9^	24 (82.8)
Memory impaired ^9^	6 (21.4)
Attention/processing speed impaired ^9^	12 (42.9)
Executive function impaired ^9^	4 (13.8)
Diagnosis regarding cognition ^1^	
No cognitive complaints	11 (22.9)
Subjective cognitive decline	17 (35.4)
Mild Cognitive Impairment	17 (35.4)
Dementia	3 (6.3)

Note. *N* = number of patients; % = percent; PFBC = Primary Familial Brain Calcification; ADL = activities of daily living; iADL = instrumental activities of daily living; ^1^ as a percentage of the total population; ^2^ as a percentage of patients who underwent genetic testing; ^3^ as a percentage of the patients with a genetic mutation; ^4^ as a percentage of the symptomatic population; ^5^ Parkinsonism was defined as a combination of bradykinesia/hypokinesia plus tremor or rigidity or both; ^6^ as a percentage of patients with motor dysfunction; ^7^ cerebellar dysfunction was defined as the presence of either limb ataxia, gait ataxia or intention tremor or a combination of these symptoms; ^8^ two patients were excluded from analysis because of performance below threshold in performance validity testing; ^9^ as a percentage of patients with cognitive disorders.

**Table 3:** Frequency and severity of calcifications per location.

Brain Area	*N* (%)	*M* ^1^	*SD*
Lentiform nucleus	48 (96.0)	7.8	2.5
Caudate nucleus	35 (70.0)	4.8	4.3
Thalamus	34 (68.0)	4.8	3.8
Subcortical white matter	32 (64.0)	3.3	3.4
Cerebral cortex	18 (36.0)	1.1	1.7
Cerebellar hemispheres	39 (78.0)	4.8	3.6
Vermis	24 (48.0)	1.3	1.7
Mesencephalon	8 (16.0)	0.7	1.9
Pons	6 (17.1)	0.3	1.0
Medulla	3 (6.0)	0.1	0.6
TCS	50 (100.0)	29.0	18.1

Note. *N* = number of patients; *M* = mean; *SD* = standard deviation; TCS = Total Calcification Score; ^1^ The maximum calcification score for the vermis, cortex, pons, and medulla is 5 per area. Bilateral areas have a maximum score of 10.

**Table 4:** Association of the total calcification score with motor dysfunction and cognitive disorders.

	Univariate Analysis			Age/Sex-Adjusted Analysis		
	OR	95%-CI	*p*-Value	OR	95%-CI	*p*-Value
**Motor dysfunction**	**1.05**	**1.01–1.09**	**0.02**	1.04	1.00–1.09	0.05
Parkinsonism	1.03	1.00–1.07	0.14	1.04	1.00–1.09	0.09
Bradykinesia/hypokinesia	**1.06**	**1.02–1.11**	**<0.01**	**1.07**	**1.02–1.12**	**<0.01**
Rest tremor	0.96	0.91–1.02	0.16	0.94	0.88–1.01	0.08
Rigidity	1.02	0.99–1.06	0.20	1.02	0.99–1.06	0.20
Cerebellar dysfunction	**1.04**	**1.00–1.08**	**0.03**	1.04	1.00–1.07	0.06
Limb ataxia	**1.04**	**1.01–1.08**	**0.03**	1.03	1.00–107	0.07
Gait ataxia	**1.05**	**1.00–1.10**	**0.04**	**1.06**	**1.00–1.12**	**0.04**
Intention tremor	1.01	0.96–1.07	0.62	1.04	0.97–1.11	0.27
Increased fall risk	**1.05**	**1.01–1.09**	**0.02**	**1.04**	**1.00–1.08**	**0.03**
**Cognitive disorders ^2^**	1.03	0.99–1.06	0.14	1.03	0.99–1.07	0.10
Global cognitive functioning (MoCA)	1.01	0.98–1.04	0.50	1.01	0.97–1.04	0.77
Memory ^3^	1.03	0.98–1.09	0.18	1.03	0.98–1.09	0.28
Attention/processing speed	1.03	1.00–1.07	0.09	**1.06**	**1.01–1.12**	**0.02**
Executive function ^3^	0.96	0.89–1.03	0.22	0.90	0.79–1.03	0.13

Note. OR = odds ratio; 95%-CI = 95% confidence interval; MoCA = Montreal Cognitive Assessment; ^2^ two patients were excluded from analysis because of performance below threshold on performance validity testing; ^3^ one patient completed less than two tests within this domain; bold values indicate significance (*p* < 0.05).

**Table 5:** Association of location of calcification with motor dysfunction and cognitive disorders.

	Univariate Analysis			Age/Sex-Adjusted Analysis		
Location of Calcification	OR	95%-CI	*p*-Value	OR	95%-CI	*p*-Value
Motor dysfunction						
Lentiform nucleus	**1.57**	**1.14–2.16**	**<0.01**	**1.50**	**1.07–2.09**	**0.02**
Caudate nucleus	1.12	0.97–1.29	0.13	1.09	0.94–1.27	0.25
Thalamus	1.08	0.92–1.25	0.36	1.05	0.89–1.23	0.56
Subcortical white matter	**1.23**	**1.00–1.51**	**0.05**	1.19	0.96–1.47	0.11
Cerebral cortex	1.31	0.89–1.95	0.17	1.22	0.81–1.84	0.35
Cerebellar hemisphere	**1.23**	**1.03–1.47**	**0.03**	1.17	0.97–1.42	0.11
Vermis	**1.62**	**1.01–2.58**	**0.04**	1.46	0.92–2.34	0.11
Midbrain	2.17	0.55–8.61	0.27	2.08	0.53–8.19	0.29
Pons	2.16	0.57–8.16	0.26	1.93	0.51–7.22	0.33
Medulla	1.48	0.36–6.14	0.59	1.32	0.28–6.17	0.73
Cognitive disorders						
Lentiform nucleus	1.13	0.89–1.43	0.32	1.16	0.89–1.52	0.29
Caudate nucleus	1.02	0.89–1.17	0.74	1.03	0.89–1.20	0.65
Thalamus	1.03	0.84–1.20	0.71	1.03	0.88–1.21	0.70
Subcortical white matter	1.21	0.99–1.48	0.06	**1.25**	**1.01–1.54**	**0.04**
Cerebral cortex	1.22	0.85–1.75	0.29	1.29	0.88–1.90	0.19
Cerebellar hemisphere	1.09	0.93–1.29	0.27	1.12	0.93–1.36	0.22
Vermis	1.29	0.88–1.88	0.19	1.44	0.94–2.22	0.10
Mesencephalon	NA	NA	NA	NA	NA	NA
Pons	1.28	0.67–2.42	0.46	1.41	0.73–2.71	0.31
Medulla	NA	NA	NA	NA	NA	NA

Note. OR = odds ratio; 95%-CI = 95% confidence interval; NA = not applicable; bold values indicate significance (*p* < 0.05).

## Data Availability

Data were generated at the geriatric outpatient clinic of the University Medical Center Utrecht, the Netherlands. Derived data supporting the findings of this study are available from the corresponding author (H.L.K.) upon request.

## Appendix A

**Table A1:** Overview of all laboratory and serological testing performed for each patient.

Laboratory Testing (Serum)		
Sodium	Potassium	Calcium
Magnesium	Phosphate	Aluminum
Hemoglobin A1C	Urea	Creatine + eGFR
Alkaline Phosphatase	Gamma-Glutamyl Transferase	ASAT
ALAT	Lactate Dehydrogenase	Creatine Kinase
Albumin	Total protein	C-Reactive Protein
Triglycerides	Cholesterol	HDL Cholesterol
LDL Cholesterol	Non-HDL cholesterol	Ferritin
Folic Acid	Vitamin B12	Glucose
Hemoglobin	Hematocrit	Erythrocytes
MCV	MCH	MCHC
Erythrocyte Sedimentation Rate	Platelets	Leukocytes
TSH	Free Thyroxine (T4)	Parathyroid Hormone
25-Hydroxy Vitamin D	Copper	Zinc
Zinc (Dissociated Serum)		
**Infectious disease serology**		
Brucella species antibodies		
Cytomegalovirus quantitative DNA PCR		
Human Immunodeficiency Virus-1/2 antibodies and p24 antigen		
Human herpesvirus type 6 and 8 DNA PCR		
Rubella virus IgM and IgG		
Toxoplasmosis gondii IgM and IgG		
Tuberculosis (using Interferon Gamma Release Assay/QuantiFERON test)		

Note. eGFR = Estimated Glomerular Filtration Rate; ASAT = Aspartate Aminotransferase; ALAT = Alanine Aminotransferase; HDL = High-Density Lipoprotein; LDL = Low-Density Lipoprotein; MCV = Mean Corpuscular Volume; MCH = Mean Corpuscular Hemoglobin; MCHC = Mean Corpuscular Hemoglobin Concentration; TSH = Thyroid-Stimulating Hormone; PCR = Polymerase Chain Reaction.
